# Report of Three Cases of Empyema

**Published:** 1909-03

**Authors:** Chas. P. Ward

**Affiliations:** Atlanta, Ga.


### REPORT OF THREE CASES OF EMPYEMA.

BY CHAS. P. WARD, M. D., ATLANTA, GA.

The radical procedure in Empyema is resection. If extensive adhesions of pleura are present they are broken up—even Estander's operation may be deemed necessary.

The methods of aspiration and irrigation that bear a relation to the method used in the cases to be reported are Potain's syphon tubes. Bulan's method, Hulton's duck-bill drainage and Pertlie's aspirator and air-pump. These devices are used to prevent the entrance of air to the pleural cavity, to preserve the integrity of the chest wall, and allow the lung to assume its normal position, if free. The irrigation of the pleural cavity, after resection is advocated by Parker, Bealz, Fernelt, Tanfilief, Lavaschaff and John Wyeth. Wyeth uses bichloride 1-1,00 to 1-3,000; Tanfilief used normal saline solution in ten cases, with rapid recovery and final cure in all. Zeman irrigated by submerging the patient in a bath of sterile water—the acts of respiration washing out the cavity.

Wm. Ewart, Samuel West and Edmund Anders advise the use of drainage first.

Any procedure that admits air to the pleural cavity may cause serious embarrassment or death, due to pleural apoplexy, shock or collapse of the lung.

The method used in the following cases differs from any I have been able to find in the liturature at my disposal, and it was evolved to meet indications in the first case.

The apparatus consists of an aspirator, a fountain syringe or irrigator, a glass "Y", or a Kashimoura's thoracic trocar in order to see that the solution is free of air, stiff tubing for connections, trocar and canula—a hot water bath (103 to 110 degrees) through which the irrigating tube passes in order to prevent chilling of the pleura—also suitable solutions for irrigating.

To one side of the "Y" the aspirator is attached, to the other side, the irrigator. The stem of the "Y" is fitted with a short piece of stiff rubber tubing that fits snugly over the canula. With the apparatus thus equipped the trocar and canula is inserted at point of election into the pleural cavity. The trocar is now withdrawn, a finger is placed over the opening in the canula to prevent entrance of air until the tube on the stem of the "Y" can be attached to the canula. Now allow suction, and if the fluid is present let the water from the irrigator flow through the "Y" into the aspirator in order to remove any air that might be present. Cut off the solution and continue mild suction of the pus until the fluid stops, or the patient shows signs of distress. Cut off suction and allow solution to flow into the chest until the quantity withdrawn is replaced, again cut off solution and allow suction till symptoms indicate the need of refilling the chest. Repeat this procedure till the wash water comes away clear—this of course if the patient stands the ordeal well.

The advantages of this procedure are: (1) In case of collapse, hot water can immediately be thrown into the chest. (2) It admits no air to the cavity during the process of irrigation. (3) The suction induced favors the descent of the lung, while in resections the atmospheric pressure is liable to cause collapse of this organ. (4) The preservation of the chest wall, for following resections there is always more or less distortion thereby encroaching upon the normal capacity of the lung. (5) This can be done under local anaesthesia, a decided advantage as such cases do not take a general anaesthetic well.

Case 1.—Charles R., age 24, family history and previous history negative.

Present history, January 15th, had pneumonia of three weeks' duration. This was followed by slow fever, weakness, loss of appetite, pains in right side, especially on taking a deep breath. This continued with cough most of the time difficulty in breathing. A diagnosis of tuberculosis was made in April. The above condition continued till June 1st, when patient began to grow worse rapidly, complaining of "stuffiness" in his right side. The cough became almost incessant, with scant expectoration—loss of weight, inability to sleep and great weakness. I first saw patient on June 15, 1906. Temperature 104; respiration 50 to 60. The right chest was bulging and the nipple area showed redness. Percussion gave flatness over entire side which extended beyond the spine on left side at base of lung.

Anscultation—Vesicular murmur absent except tubular breathing over larger bronchi which were forced high up into the apex. Diagnosis—Empyema. Immediate aspiration drew off two full quarts of pus. Next day patient was brought to city for operation and 60 oz. drawn away. This was followed by a chill with temperature 101 to 105. On June 20th, one quart was removed before introducing any solutions and I estimate that fully one pint came away with the wash water. The apparatus did not work so well this time because of a too soft tube which would collapse at sterile, normal saline solution leaving 24 to 30 oz. in the chest. at this time. On the 24th I aspirated again drawing off 24 oz. pus before irrigating. The patient being stronger I washed the chest thoroughly with 3 quarts bi-chloride 1-4,000, followed by 3 quarts sterile, normal asline solution leaving 24 to 30 oz. in the chest. There was in this water fully 20 oz. of pus making about 13 or 14 pints of pus from this case in 11 days.

The patient improved rapidly under tonics and food, and the fluid was gradually absorbed.

In April, 1907, physical examination showed normal respiration over entire lung—a few bands of adhesion and thickening of pleura. This man is sound today with no evidence of ever having had this trouble.

Case 2.—Miss S. W. L., age 30. Family history negative. She nursed an aunt 65 years of age said to have bronchitis. Previous history—Had cough with pain in left side about a year ago, attack lasted several months. Had coughed occasionally since.

Present history.—About January 1st, 1907, she was exposed to cold with recurrence of pain in side and cough. No expectoration, general malaise and loss of appetite. I first saw patient January 19. Temperature 103, pulse 120 weak. History of having had fever for over a week. Examination of chest revealed friction sounds over posterior scapulary space and around to nipple line—otherwise negative. Tongue was coated, bowels constipated, headache in the evening and some distention over abdomen. Her temperature ranged from 101 to 104 during the next eight days. At the end of this time physical examination showed fluid in left chest extending to eighth rib scapular line. Put patient on iodides, with restricted fluids. Temperature still continued typical typhoid or septic course. Six days later physical examination showed increased effusion, and aspiration advised. On February 5th I drew off 12 oz. sero-purulent fluid before irrigating with 1-8,000 bi-chloride followed by 3 quarts saline solution leaving about 10 oz. in cavity. I estimate 24 cz. of sero-purulent fluid in this case—negative by culture. The sputum showed positive T. B. Temperature declined and was normal on fifth day—the fluid disappeared and patient was able to return to her home in the mountains February 25th, but her convalescence was slow. She grew very weak during that summer and sputum showed active T. B. Case followed, and following result reported by letter from her on February 1st, 1909. Patient went to Asheville, N. C., and took tuberculin, remaining there for six months. She returned to Asheville last summer, and at present weighs 145 lbs.—more than every before. She believes that the continued tuberculin immunity will prove a cure for her.

Case 3.—Mrs. F. M. Y., age 45. Family history obscure. Previous history—has had "womb trouble" for years.

On March 28, 1907, patient brought to my office from home in Florida—said to have hopeless tuberculosis. She was on her way to North Georgia to die, so she said. She gave history of pain in right side with cough for past 3 months. Temperature 100, pulse 112; respiration 34. Examination indicated fluid. I aspirated three days later and drew off 30 oz. sero-purulent fluid which was negative microscopically and by culture. Irrigated thoroughly as in the other cases, leaving about 10 oz. of saline in the chest. Estimated 12 oz. fluid to have come away with wash water. Patient made rapid recovery, fluid being rapidly absorbed. On third day temperature and pulse normal and eight days after operation she was carried home much improved. I have examined this case three times since finding the vessicular murmur normal over entire lung with few friction rales indicating some pleural adhesions.

